# A Review on Citric Acid as Green Modifying Agent and Binder for Wood

**DOI:** 10.3390/polym12081692

**Published:** 2020-07-29

**Authors:** Seng Hua Lee, Paridah Md Tahir, Wei Chen Lum, Li Peng Tan, Paiman Bawon, Byung-Dae Park, Syeed SaifulAzry Osman Al Edrus, Ummi Hani Abdullah

**Affiliations:** 1 Institute of Tropical Forestry and Forest Products (INTROP), Universiti Putra Malaysia, Serdang 43400 UPM, Selangor, Malaysia; parida@upm.edu.my (P.M.T.); saifulazry@upm.edu.my (S.S.O.A.E.); 2 Institute for Infrastructure Engineering and Sustainable Management (IIESM), Universiti Teknologi MARA, Shah Alam 40450, Selangor, Malaysia; lumweichen@outlook.com; 3 Faculty of Veterinary Medicine, Universiti Malaysia Kelantan, Pengkalan Chepa, Kota Bharu 16100, Kelantan, Malaysia; 4 Faculty of Forestry and Environment, Universiti Putra Malaysia, Serdang 43400 UPM, Selangor, Malaysia; ummi@upm.edu.my; 5 Department of Wood and Paper Science, Kyungpook National University, Daegu 41566, Korea; byungdae@knu.ac.kr

**Keywords:** citric acid, esterification, binding agent, wood modification, wood composite

## Abstract

Citric acid (CA) can be found naturally in fruits and vegetables, particularly citrus fruit. CA is widely used in many fields but its usage as a green modifying agent and binder for wood is barely addressed. Esterification is one of the most common chemical reactions applied in wood modification. CA contains three carboxyl groups, making it possible to attain at least two esterification reactions that are required for crosslinking when reacting with the hydroxyl groups of the cell wall polymers. In addition, the reaction could form ester linkages to bring adhesivity and good bonding characteristics, and therefore CA could be used as wood binder too. This paper presents a review concerning the usage of CA as a wood modifying agent and binder. For wood modification, the reaction mechanism between wood and CA and the pros and cons of using CA are discussed. CA and its combination with various reactants and their respective optimum parameters are also compiled in this paper. As for the major wood bonding component, the bonding mechanism and types of wood composites bonded with CA are presented. The best working conditions for the CA in the fabrication of wood-based panels are discussed. In addition, the environmental impacts and future outlook of CA-treated wood and bonded composite are also considered.

## 1. Introduction

**Figure 1:**
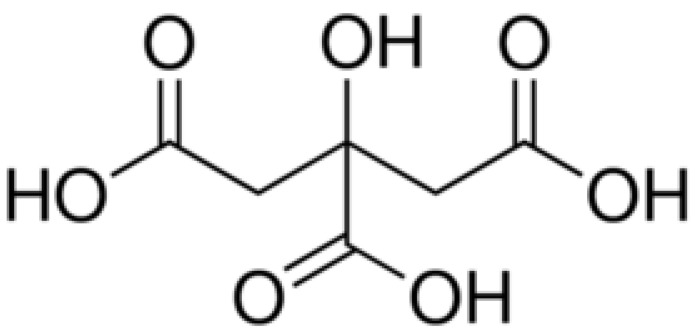
Chemical structure of citric acid.

Citric acid (CA) is an organic acid with the formula C_6_H_8_O_7_, as shown in Figure 1. Owing to the fact that the anion can be stabilized by intramolecular hydrogen-bonding from other protic groups on CA, CA is a slightly stronger acid than typical carboxylic acids [1]. CA is also called different names, such as 2-hydroxy-1,2,3-propanetricarboxylic acid, β-hydroxytricarballyic acid, 3-carboxy-3-hydroxypentanedioic acid, and 3-carboxy-3-hydroxypentane-1,5-dioic acid [2]. CA could be found naturally from a variety of fruits and vegetables, particularly citrus fruits such as oranges, tangerines, lemons, limes, and pomelos. Penniston et al. [3] stated that lemons and limes are among the citrus fruits that contain a higher concentration of CA, which amount to 8% of the dry weight of the fruits.

CA is odourless and exists in white coloured crystalline powder form at room temperature. Moreover, CA also exists in two other forms, namely anhydrous and monohydrate forms. The former is crystallized from hot water while the latter from cold water. Both forms have different molar masses, namely 192.123 g/mol and 210.14 g/mol, respectively. The density of CA in both forms are different too, whereas anhydrous has a density of 1.665 g/cm^3^, the density for monohydrate is 1.542 g/cm^3^. The melting point of CA is 156 °C. At 175 °C, CA starts to decompose through the loss of carbon dioxide and water [1].

The discovery of CA could be traced back to eighth century. Abu Musa Jabir ibn Hayyan, also known as Geber, was a renowned Islamist alchemist in the eighth century. He was credited for the first discovery of CA, the sour component of lemons and other unripe fruits [4]. In 1923, the Currie-Thom fermentation process discovered by food chemist James Currie and microbiologist Charles Thom was commercialized.

CA is widely used as a pharmaceutically active substance in pharmaceuticals, personal care, and cosmetic products, as an acidulant and pH stabilizer in beverages, blood anticoagulants, as well as diuretic and flavoring agents [5]. In recent years, many emerging uses of CA have also been identified, e.g., crosslinker, disinfectant, environmental remediation, and extracting agent [5]. However, despite the versatility of CA, the reports on its usage as a wood modifying agent and wood adhesive are relatively scarce. As a major aminoplastic resin that is prevalently used for the manufacturing of wood-based composites, urea formaldehyde (UF) resin is claimed as the main source of formaldehyde emitted indoors [6]. The continuous emission of formaldehyde will subsequently lead to a medical condition called sick house syndrome, whereby the occupant experiences various symptoms such as headache, nose and throat irritation, and fatigue [7]. Therefore, many countries have already imposed a more stringent rule in limiting the level of emissions. On account of that, the application of a non-formaldehyde-based resin or green binder such as CA could be a partial solution for the issue. In the recent years, there are many studies dealing with the synthesis of environmentally friendly wood adhesives in response to environmental protection. The green wood adhesives include glyoxalated corn stalk lignin-based wood adhesive [8], sucrose and ammonium dihydrogen phosphate (ADP) adhesive [9,10], defatted soybean flour-based wood adhesive [11], tannin–sucrose adhesive [12], and many other types of wood adhesive.

A brief review regarding the application of CA as green binder and impregnant for wood has been compiled by Chayono and Syahidah [13]. The authors reviewed the potential of CA to serve as a binding agent for lignocellulosic materials. The authors reported that, with the addition of 20% CA, wood wastes as well as non-woody materials could be fabricated into particleboard with good performance. The authors also revealed that the pressing temperature of 180 °C is the optimal temperature, resulting in superior bonding strength and improving along with increasing temperature. The authors also found that the density increment for wood impregnated with CA is lower than that of phenol formaldehyde (PF)-bonded wood. This might be due to the fact that PF resin are filled in the lumen while CA is expected to form ester bond in the cell wall. In a chapter by He and Umemure [2], the authors reviewed the applied research and commercial production of CA. Application of CA as cross-linking catalyst, a cross-linking agent, or a dispersing agent in improving the strength of the adhesive as well as serving as main bonding agent has been discussed. The authors attributed the adhesiveness of CA to the formation of ester linkages between CA and lignocellulose molecules. However, the authors also recommended that the processing parameters should be improved. Overall, the authors have pointed out the potential of CA to serve as renewable natural adhesive for wood and lignocellulosic materials. The literature referred to in the chapter were published prior to 2016. Therefore, an up-to-date review was prepared in this paper with the emphasis put on the application of CA as a major bonding agent for wood-based composites. In addition, the usage of CA as a modifying agent for wood has also been discussed in the present paper.

## 2. Esterification for Wood Modification

Owing to the persistent environmental issue, more new green modification technologies of wood has been introduced in hope to replace the conventional preservative treatment methods [14]. The main purpose for wood modification is to permanently change the molecular structure of the cell wall polymers and resulted in wood with improved properties. The examples of wood modification method include heat treatment, acetylation, furfurylation and resin treatment [15,16]. Esterification is one of the green wood treatment methods. Esterification is a chemical reaction between acid (carboxylic acid) and alcohol (or other -OH) to form ester and water. Wood esterification is one of the most common chemical reaction that applied in wood modification. It is a reaction that can be performed within the wood cellular structure and therefore is appropriate for wood modification [17]. There are two types of wood modification adopting the esterification process, namely wood esterification and esterification-based bulk impregnation [18]. The former involves a reaction between the functional groups of a treating agent with hydroxyl groups of wood. Meanwhile, the latter involves the reaction of the functional groups of one treating agent and the hydroxyl groups from other treating agents and/or wood [19]. Esterification with CA is an inexpensive and environmentally friendly wood modification method to bestow good dimensional stability as well as biological durability to wood and lengthen its service life [20].

In the mid-1980s, non-formaldehyde-based finishes, namely 1,2,3,4-butanetetracarboxylic acid, (BTCA) was discovered to be a promising crosslinking agent for cotton cellulose [21]. As cotton cellulose behaves highly similar to wood polysaccharides, it has been deduced that polycarboxylic acids could act as a treating agent in wood modification [22]. The process is called esterification. Simply put, the mechanism involved in the formation of anhydride and the formed cyclic anhydride was then reacted with the hydroxyl group of the wood polysaccharides to form ester linkages [23]. Wood modification using aromatic polycarboxylic acid compound is relatively new as the first study was reported by Peyer et al. [24], where a polycarboxylic acid resin was used to soak the flakes of Aspen wood. The dimensional stability improved significantly. On the other hand, the moisture and swelling of small wooden blocks was reduced when treated with CA and BTCA as reported by Katovic et al. [25].

### 2.1. Reaction Mechanism of Interaction between Wood and Citric Acid

The reaction mechanism between wood and CA involved a two-step esterification where anhydride was formed first, followed by the reaction of this cyclic anhydride with hydroxyl groups of wood and formed ester linkages [22,26]. Decomposition of CA to unsaturated acids happen when it was heated at 175 °C. Acetone dicarboxylic acid, aconitic acid and itaconic acid are the main products that are generated through dehydration or decarboxylation [26]. When subjected to further heating, methyl maleic anhydride is formed [27].

In a study by Fang et al. [28], quantitative analysis of the cross-linking reaction process and reaction parameters (esterification degree, carbonyl ratio, cross-linking degree) of polycarboxylic acid and wood components by FTIR proves that the cross-linking esterification reaction process of polycarboxylic acid and wood is as follows: First, the hydroxyl groups of the carboxylic groups in the polycarboxylic acid are dehydrated to form an anhydride, and then the hydroxyl groups of the wood and the anhydride undergo a nucleophilic substitution reaction to form an ester. It was inferred that the crosslinking reactions between polycarboxylic acid and wood went through cyclic anhydride intermediates, rather than undergoing a nucleophilic addition of the hydroxyl group and the carbonyl group.

### 2.2. Citric Acid Compared to Other Polycarboxylic Acids

CA that contains three –COOH groups is a tricarboxylic acid. First of all, polycarboxylic acids used in wood esterification must have three or more acid groups that are able to react with the hydroxyl groups of the cell wall polymers. Maleic acid and succinic acid having two acid groups could be adopted theoretically. Nevertheless, it is very hard to attain a second esterification reaction that is required for crosslinking [29]. On the other hand, larger acids such as BTCA are able to crosslink to a greater extent due to their ability to form simultaneous anhydride intermediates [30].

However, in common practice, BTCA and CA are the two most used polycarboxylic acids as crosslinking agent for cotton cellulose. BTCA and CA are known to show the possibility to react with wood cell wall polymers [22,31]. Compared to CA, BTCA could perform more effectively owing to its higher COOH group content [32,33]. Vukusic et al. [22] reported that, between CA and BCTA, BCTA was found to have a considerably higher portion bonded to wood than CA, mainly due to the fact that CA could only form two ester linkages with cellulose. Furthermore, Yang et al. [34] reported that there is an interference between the α-hydroxyl group of CA and wood cellulose, which might also contribute to the lower amount of CA bound to wood. However, CA is cheaper as an easily accessible natural or synthetic product [26]. In addition, Vukusic et al. [22] treated fir and beech wood with BTCA and CA and found that the tensile retention strength of the CA-treated samples was better than that of the BCTA-treated samples. Therefore, many researchers have adopted CA as a wood modifying agent as well as wood binder.

### 2.3. Citric Acid for Wood Modification

**Figure 2:**
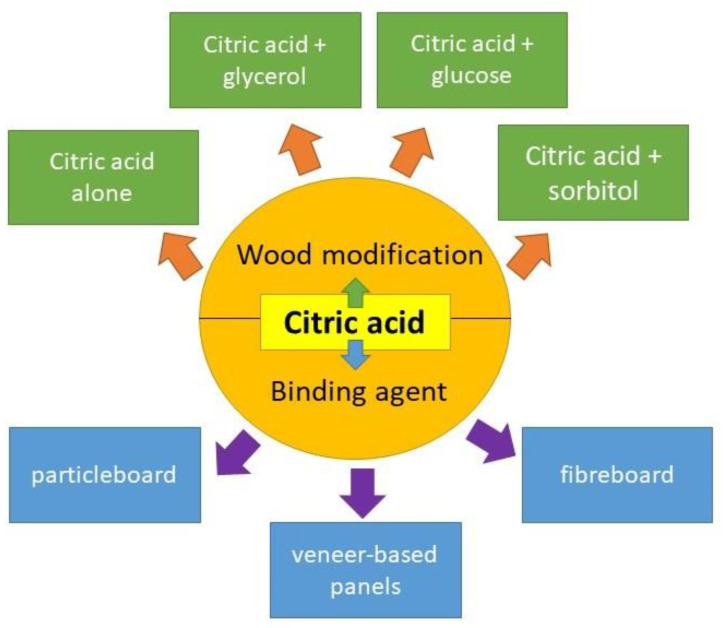
Citric acid as wood modifying agent and binding agent for wood composite.

Figure 2 shows the role of the CA as wood modifying agent and binding agent for wood composite. In the first part of this review, the application of CA as a wood modifying agent are discussed. In the second part, CA as a major binding agent for wood composites such as particleboard, fibreboard, and veneer-based panels are addressed.

Generally, the modification of wood using CA could result in several advantages and disadvantages to the treated wood as shown in Table 1. In wood modification, CA could be used alone or added with other reactants to enhance its effectiveness. The reactants employed include glycerol, glucose, and sorbitol.

#### 2.3.1. Citric Acid Alone

Vukusic et al. [22] compared the effectiveness of 1, 3-dimethylol 4, 5-dihydroxy ethylene urea (DMDHEU) and CA on the tensile strength and dimensional stability of fir and beech wood. The results revealed that treatment with CA resulted in higher anti swelling efficiency (ASE) compared to that wood samples treated with DMDHEU. In addition, reduction in WA was also higher for CA-treated wood. As for tensile strength, DMDHEU treated wood showed lower tensile strength retention compared to CA and BCTA treated wood.

Šefc et al. [35] modified fir and beech wood with CA and the compression strength of the modified wood were investigated. The average compression strength of the modified wood improved significantly in the case of fir wood. However, no significant improvement in beech wood was observed. In this same study, the authors also employed two different curing methods, namely convection oven heating and microwave curing. However, no improvement was observed when microwave curing was employed despite its elaborated advantages by several researchers. However, modification of wood with CA did not improve the weathering resistance of modified wood as reported by Miklečić and Jirouš-Rajković [36]. This can be explained by the fact that CA only modifies cellulose but not lignin. Weathering degradation often occurs on the lignin polymer photochemically by exposure to UV light. As a result, the cellulose fibres are loosened and can be easily washed off from the wood surface, which subsequently leads to a rough surface of the weathered wood samples.

Tarasin & Rattanapun [37] treated *Melaleuca cajuputi* wood with CA using dipping and vacuum methods and the resistance of the treated wood against termite, *Coptotermes gestroi* Wasmann, was evaluated. Both dipping and vacuum methods imparted the wood with higher resistance against termites. Toyoshima et al. [38] stated that the CA might have affected the metabolism of termites and subsequently led to higher mortality of termites. On the other hand, the improvement in termite resistance might also be caused by the strong odour emitted by CA and repelled the termites from approaching the wood samples [37]. Interestingly, wood samples treated with 3% CA using vacuum method performed similar to that of samples treated with 1.5% boron using the same method.

Reports on the effects of CA treatment on the mechanical properties of wood are rather limited. Treatment with CA has improved the modulus of elasticity (MOE) of the treated as a result of cell wall bulking and crosslinking of cell wall polymers [39]. Apart from that, compression strength was also improved. However, CA treatment increased the brittleness of treated wood as a 50% loss in impact strength was recorded, with or without the addition of sodium hypophosphite (SHP) [40]. A reduction in MOR was also recorded.

#### 2.3.2. Citric Acid with Glycerol

In wood modification, CA can be combined with glycerol, a simple polyol compound generated from the biodiesel industry as a by-product, to attain better treatment efficiency [17,41]. These studies have shown that the polymer of glycerol and CA could serve as a promising treating agent for wood. With the existence of heat, both reactants can react and form a three-dimensional polymeric structure [17]. Berube et al. [17] stated that unreacted carboxylic groups from the glycerol-based polymer are able to bond with free hydroxyl groups from wood macromolecules. Consequently, the polymers could be retained in the cell wall after a curing and leaching test owing to the formed chemical bond.

L’Hostis et al. [41] treated beech wood with CA and tartaric acid (TA) alone and the combination of CA with glycerol and TA with glycerol. In the case of CA, CA+glycerol has slightly higher impregnation rate compared to CA alone. Contrarily, impregnation rate of TA decreased after combined with glycerol. Further, the addition of glycerol improved the leaching resistance of CA and TA which was mainly due to the in-situ copolymerization of glycerol that supported fixation in the wood samples. In comparison, the leaching resistance of CA was higher than TA, where a heating temperature of 120 °C provides sufficient leaching resistance to the combination of CA and glycerol while TA and glycerol requires a heating temperature of 140 °C to attain the same effect. Improvement in leaching resistance could be attributed to grafting via ester bonds and the formation of an insoluble polyester in the cell wall. According to Halpern et al. [42], these reactions are heat catalyzed and therefore occurred only at high heating temperature. Bending resistance of beech wood treated with CA and TA combined with glycerol was also better compared to that of samples treated with CA and TA alone due to higher amount of formed polyester in the cell wall. Decay resistance against *Coriolus versicolor* also saw a better trend in CA+glycerol compared to TA+glycerol. CA performed better than TA was basically owing to fact that CA has one extra functional group than TA.

#### 2.3.3. Citric Acid with Glucose

Glucose crosslinked with CA could also be used in wood modification as it is an aldose sugar that are able to react with the hydroxyl groups of wood [43]. Owing to its small molecular mass of 180 Dalton, glucose can be easily penetrated into wood cell wall. However, the low fixation ratio of glucose is a problem that obsessed by researchers [44]. Water leaching was very high, even when the glucose was oxidized using Fenton’s reagent to improve its fixation [44]. However, He et al. [45] reported that CA could serve as a crosslinker between glucose and wood cell wall polymers and promote the fixation ratio of glucose in wood.

He et al. [45] reported that there was no weight percent gain after leaching recorded when wood was impregnation with glucose only. Guo et al. [46] also reported an almost total loss of deposition on glucose-treated Scots pine after water leaching. However, after being activated with Fenton reagent, the leaching ratio decreased as the aldehyde functionality of glucose and wood hydroxyl groups formed stable acetal bonds under catalysis with MgCl_2_ and high temperatures [47]. Scots pine treated with CA has relatively lower leaching ratio. However, the leaching ratio increased when activated glucose was added into CA due to the dilution effect of on CA.

Guo et al. [46] discovered that the effect of fixation is greater at lower activated glucose concentration, i.e., 4.5 wt % activated glucose + 8.2 wt % CA. He et al. [45] explained that this phenomenon was a result of CA acts as crosslinker between wood polymers and activated carbon that containing hydroxyl groups. Furthermore, the low pH value of the activated glucose and CA solution has promoted its fixation in wood. Although the application of activated glucose in wood modification did not lead to satisfactory results, it does, however, impart better impact strength to the modified wood. Treatment with CA often resulted in embrittlement of the treated wood as a result of crosslinking within the cell wall matrix [40]. Consequently, the impact strength of modified wood was reduced. When combined with CA, the impact strength of the modified wood increased along with increasing activated glucose concentration. Activated glucose consumed a high amount of CA and therefore the crosslinking of the cell wall by CA was reduced as well as the extent of embrittlement [46].

#### 2.3.4. Citric Acid with Sorbitol

Sorbitol is a cheap feedstock chemical generated commercially from starch and cellulose [48,49]. Sorbitol is able to react with CA to form polyester at high temperatures, making it suitable for use to modify wood [50]. However, reports on the use of sorbitol as wood modification agent are rather limited. The earliest report was authored by Kiljunen et al. [51], which is a patent reported on the application of sorbitol and CA as well as other combinations of water-soluble reagents in treating wood samples.

European beech (*Fagus sylvatica*) sapwood modified with CA and sorbitol solution at different concentrations has shown an increment in density, pH, and thickness swelling (TS), where the increment increased along with increasing CA-sorbitol concentrations [19]. Larnøy et al. [52] treated Scots pine sapwood (*Pinus sylvestris*) with a combination of sorbitol and CA and some selected properties of the treated wood were assessed. As a result of the treatment, resistance against brown rot, white rot, and blue stain fungi of the treated pine sapwood were improved significantly. Beck [53] prepared a solution of CA to sorbitol with a 3:1 molar ratio and used the mixture to modify Scots pine wood. The results confirmed that the treatment is promising to impart decay resistance to the modified. However, the curing parameters used in the study had to be improved to overcome the leaching issue.

### 2.4. Factor That Affect the Effectiveness of Wood Modification by Citric Acid

There are several factors that affect the effectiveness of the wood modification using CA include heating temperature, modification period, and catalyst type and wood species [54].

#### 2.4.1. Catalyst Type

A variety of catalysts could be used to facilitate the esterification reaction where SHP is known as the most efficient one [40]. The rate of formation of anhydride intermediates was enhanced by the addition of SHP in addition to the minimized decomposition of cellulose during the curing process. In fact, without SHP, the application of CA alone could react with wood. According to the study by Feng et al. [40], a weight percent gain (WPG) of 4.6% was recorded in CA-treated poplar wood with SHP addition after water leaching. The WPG increased along with increasing CA concentration. Apparently, addition of SHP did improve the WPG. The WPG increased along with addition dosage of SHP and reached a maximum WPG of 6.3% at 10% SHP addition. However, Feng et al. [40] concluded that 5% addition is the most economical.

SHP (NaH_2_PO_2_) is generally recognized as the best catalyst for PCA as reported in several studies [22,55]. Šefc et al. [54] modified fir and beech wood with CA added with different catalysts, namely SHP (NaH_2_PO_2_) and monosodium phosphate (NaH_2_PO_4_). Nevertheless, the author reported that there is no significant difference between these two catalysts in terms of ASE. To the favour of NaH_2_PO_4_, it is cheaper than NaH_2_PO_2_.

Berube et al. [17] compared the effectiveness of four different catalysts incorporated into the mixture of CA and glycerol in treating white pine and lodgepole pine. The catalysts used are chlorhydric acid (HCl), para-toluenesulphonic acid (p-TSA), phosphotungstic acid (TPA) and iron(II) sulphate (FeSO_4_·7H_2_O). The results revealed that HCI and p-TSA are more effective as they showed higher carboxyl conversion to ester. Furthermore, HCI and p-TSA are also more thermally stable as they displayed higher residue mass. However, p-TSA was found to be more effective in treating white pine as a relative higher ASE value was recorded. On the contrary, HCI display equal effectiveness in treating both white pine and lodgepole pine samples.

#### 2.4.2. Wood Species

In a study by Berube et al. [17], white pine attained higher weight percent gain compared to lodgepole pine after treated with a mixture of CA and glycerol using identical parameters. The author has attributed this observation to the anatomy of the wood where lodgepole pine has lower permeability than white pine. Study by Vukusic et al. [22] also demonstrated that fir wood attained higher anti-swelling efficiency than beech wood when treated with 6.9% CA. Wood anatomy plays an important role in deciding the effectiveness of the treatment as it is directly proportional to the uptake of CA [56].

#### 2.4.3. Curing Temperature

Larnøy et al. [52] found the treated pine sapwood samples cured at 140 °C are more stable to that of cured at 103 °C as no leachate was detected in the former by HPLC analysis. It is implied that curing temperature of 103 °C is not sufficient to ensure complete reaction between CA and sorbitol. In addition, samples that cured at 140 °C attained better dimensional stability and bulking coefficient. Leaching analysis indicated that treated wood samples cured at 160 °C resulted in the lowest amount of leaching compared to that of cured at 140 °C, suggested that intensive polyesterification had occurred at this curing temperature. Doll et al. [44] and Larnøy et al. [52] suggested that 150 °C and 140 °C is a better curing temperature than other lower temperatures used in their respective studies, thus it could be concluded that temperature of at least 140 °C is required to ensure the polyesterification occurs to a greater extent. However, the curing temperature should be limited below the temperature that could cause irreversible damage to the wood samples itself.

## 3. Citric Acid as Main Bonding Component for Wood Composites

Declining use of non-renewable fossil resources is anticipated to restrict the usage of conventional synthetic resins in the near future. In addition, conventional synthetic resins such as UF resin release formaldehyde that are detrimental to human health. Several countries in the world have already imposed stringent regulation in limiting the formaldehyde emission from the wood-based panels. A non-formaldehyde-based resin or green binder are therefore in high demand. On account to that, CA, also called 2-hydroxy-1,2,3-propanetricarboxylic acid, is potentially to be applied as a green binding agent to produce particleboard with no formaldehyde emission.

### 3.1. Bonding Mechanism

When using CA as a wood adhesive, the bonding mechanism is based on the hypothesis that the esterification of wood’s hydroxyl groups by CA has formed several cross-links across the bonding interfaces. Umemura et al. [57] suggested the reaction between carboxyl groups of CA and hydroxyl groups of wood constituents formed ester linkages that resulted in adhesivity and good bonding properties. The hypothesis has been proven by Del Menezzi et al. [58] in a latter study. By using CP MAS ^13^C NMR spectra, the reaction of wood with CA at 60 °C and 180 °C was investigated by Del Menezzi et al. [58]. Phenolic hydroxyl groups of guaiacyl type lignin were found to react with CA. Similar to the wood polysaccharides, a hydroxyl group on the aliphatic chains of lignin units displays alcohol-like behaviour and therefore could react with CA too. The spectra also showed that the peak belonging to -CH_2_OH of the aliphatic side chains of lignin and of cellulose was decreased significantly, suggesting that esterification has occurred. In addition, the reduction in CH_2_OH groups may also implying the internal rearrangement of lignin, which subsequently contributing to the improvement in water resistance [59]. MALDI ToF analysis has also confirmed the co-reaction of CA with lignin and carbohydrates of wood.

### 3.2. Wood-Based Composites

From the literatures, it has been identified that CA has been used as main bonding agent for four types of wood-based composites, namely wood-based molding, particleboard, fibreboard, and veneer-based panels (plywood and laminated veneer lumber). Among them, particleboard is the most extensively studied. Table 2 listed the types of material and composites bonded with CA.

#### 3.2.1. Wood Based Molding

The application of CA as main bonding agent for wood was first investigated by Umemura et al. [57] by fabrication of bark moldings from *Acacia mangium* bark power. During the fabrication of the bark molding, the authors examined the effects of CA: bark matter ratios on its performance. The CA: bark matter ratios selected were 0:100, 11.1:88.9, 20:80, 33.3:66.7, and 40:60. Thereby, 100% bark-only moldings exhibited an extremely low MOR value of 1.5 MPa, which indicated no bonding strength was ever existed. However, the MOR value increased drastically when CA was added. With addition of 20 wt % CA, maximum MOR value of 23.4 MPa was recorded. Nevertheless, beyond 20 wt %, MOR of the bark moldings started to decrease. MOE showed the similar trend as in MOR. Based on this finding, the author confirmed the adhesivity of CA in acting as a major wood bonding agent and the optimum content for CA was 20 wt %. In addition, CA was also reported to have higher water resistance. Umemura et al. [57] concluded that the superior physical and mechanical properties were brought by the adhesiveness of CA due to the existence of ester linkages between carboxyl groups derived from CA and hydroxyl groups of the bark component.

This very first report of CA as major wood bonding agent has been used as a basis for several subsequent studies. Umemura et al. [60], on the other hand, confirmed that 20 wt % CA content was the most optimum to obtain the best mechanical strength for wood-based molding. Unfortunately, thermal properties of the wood-based molding decreased along with increasing CA content. Once again, it was clarified that the bonding mechanism of the wood-based molding was contributed by the ester linkages between wood and CA.

#### 3.2.2. Particleboard

Particleboard is the most extensively explored wood-based panel using CA as a binding agent. In the fabrication of particleboard, CA was used by Umemura et al. [63] as a binding agent, with addition of sucrose. The application of sucrose was intended to improve the bonding performance as the adhesion area of particles in particleboard production is much smaller than that of the wood flour used in the fabrication of wood-based molding. Widyorini et al. [61] fabricated particleboard from teak wood particles bonded with CA and sucrose. The addition of CA reduce the TS and WA significantly compared to sucrose bonded and binderless particleboards manufactured. CA did not adversely affect the MOR and MOE. With pressing temperature of 200 °C, CA-bonded particleboards displayed the highest MOR (12.3 MPa) and MOE (3.8 GPa).

There are several factors that influence the properties of CA-bonded particleboard, such as board density, pressing time and temperature, pre-drying treatment, and CA content [62,64,66,67,71]. Table 3 summarizes the factors that affect the properties of the resultant particleboard.

Based on the studies summarized in Table 3, it can be concluded that the effective processing conditions for CA-bonded particleboard are:

1. Pressing temperature of more than 180 °C for 10 min
2. Board density of 800 kg/m^3^
3. CA content of 20 wt % or above
4. Pre-drying treatment of 12 h at 80 °C is preferential

CA-bonded particleboard fabricated with this effective processing conditions are likely to obtain superior bending and IB strength, good dimensional stability and biological resistance and extremely low formaldehyde emission. Unfortunately, screw holding power and wet bending are under par. Another challenge facing by CA is that it required higher pressing temperature and longer pressing time than that of particleboard produced industrially, which is 140–180 °C for 5–7 min [67].

A pressing temperature of 180 °C (preferable 200 °C) is required in pressing CA-bonded particleboard as the melting point of CA is around 150 °C. At high pressing temperature (>180 °C), the core layer of the particleboard might have attained an effective temperature of more than 150 °C to cure the CA [67]. However, the pressing temperature should not exceed 200 °C as hemicellulose started to decompose at that temperature [78]. As a result, volatile components are formed from the degradation of hemicellulose and subsequently interfered the adhesiveness of the core layer. Pre-drying treatment is preferable as the 20 wt % CA often lead to high moisture of the sprayed particles. Kelly [79] stated the excessive moisture inhibits the effective reaction between the adhesive and lignocellulosic materials and hence resulted in poor bonding.

In addition to sucrose, starch could also be incorporated into CA to improve the performance of particleboard by Widyorini et al. [65]. Corn, maranta and canna starch was used in the study at mixing ratios of 100:0, 87.5:12.5 and 75:25 (CA/starch). Due to the insolubility of starch in water, particleboard with higher starch content had higher TS value. However, no significant difference was detected for WA at any mixing ratio. Among the starch used, maranta starch with the highest amylose content displayed the best dimensional stability compared to the other two types of starch. On the other hand, addition of 25% starch has improved the MOR and MOE of the particleboard. Reddy and Yang [80] attributed the improvement to the hydroxyl group in starch that can be reacted with carboxyl groups from CA. It is interesting to note that the addition of starch decreased the screw holding strength of particleboard.

CA also exhibited promising performance in bonding non-wood materials such as sweet sorghum bagasse, bamboo, sugarcane bagasse, alang-alang (*Imperata cylindrica*) and new giant reed. Nipa fronds were used in the fabrication of CA-bonded particleboard [69,70]. The author has mixed maltodextrin, a starch-based polysaccharide, with the CA and used as binder. The boards produced were lighter in colour when the amount of maltodextrin is higher. However, at a pressing temperature of 180 °C, boards bonded with 12.5% CA and higher have surpassed the requirements stipulated in JIS A 5908 in terms of TS while boards bended with 100% maltodextrin failed to meet the requirement. Ferrandez-Garcia et al. [72] fabricated particleboard from new giant reed (*Arundo Donax* L.) bonded with 10% CA. The results revealed that the produced particleboard is fit to be applied as insulating materials based on their good mechanical and thermal performance. Liao et al. [73] manufactured low density sugarcane bagasse particleboard with CA and sucrose and the particleboard showed good performance as a thermal insulator.

#### 3.2.3. Fibreboard

Indrayani et al. [74] fabricated medium density fiberboard (MDF) from pineapple leaves fibers. The pineapple leaves fibers were treated with 100% CA and a combination of 25% CA + 75% sucrose and then consolidated and hot-pressed into MDF. The produced MDF were then exposed to subterranean termites, *Coptotermes formosanus* Shiraki, for three weeks. The results revealed that pineapple leaves fibers treated with CA alone has higher resistance against termites, indicated that CA might possess some inhibitory properties against termite due to its acidity. For instance, orange oil was found to exert some extents of effectiveness against subterranean termites [81]. Widyorini et al. [75] fabricated fiberboard from elephant dung fibers bonded with CA at adhesive content of 10, 20 and 30 wt %. Using 30 wt % and a pressing temperature of 180 °C, the TS of the produced fiberboard has met the requirements stipulated in JIS A 5905 where the maximum TS was not exceeding 12%. However, when the pressing temperature was raised to 200 °C, 20 wt % CA was found sufficient to achieve the requirement. WA of the CA-bonded fiberboard was also improved along with increasing CA content. Apart from that, CA-bonded fiberboard also showed satisfactory MOR, MOE, and internal bonding strength.

#### 3.2.4. Veneer-Based Panels

The application of CA in bonding particleboard and fiberboard has seen an increasing trend since the first report by Umemura et al. [60]. However, studies on the CA-bonded wood veneers, such as laminated veneer lumber (LVL) and plywood are relatively scarce as bonding the flat surfaces are a completely different application compared to particleboard and fiberboard. The first report on the application of CA as binding agent for LVL was reported by Del Menezzi et al. [58]. Poplar veneers were modified with 20% CA and then pressed into LVL. The results suggested that CA is a promising binding agents for bonding wood veneers panels.

On the other hand, using CA as bonding agent for plywood is relatively new application as the only 2 literatures were found in the year of 2019 by Sun et al. [77] and Zhao et al. [76]. It was said that the low viscosity and solid content of CA was the main reason that restricted its use in bonding plywood. However, to overcome this barrier, a novel CA/sucrose adhesive with sufficient viscosity and solid content was synthesized by Sun et al. [77] and Zhao et al. [76] to produce plywood with good bonding performance. Sun et al. [77] synthesized CA adhesive with the addition of sucrose at different ratios and used it to bond plywood from poplar veneer. The author reported that the wet shear strength of the plywood increased with increasing CA content. The best shear strength was recorded in the plywood bonded with 25% sucrose and 75% CA, as well as increased wood failure percentage. Plywood bonded with 100% CA also showed some extent of bonding strength and wood failure compared to the plywood bonded with higher sucrose content. Sun et al. [77] has also investigated the optimum synthesis temperature and time for sucrose/CA adhesive and found that synthesis temperature of 100 °C for 2 h led to the best result.

As a continuation to the study by Sun et al. [77], Zhao et al. [76] studied the optimum pressing time and temperature and spread rate to produce plywood bonded with CA/sucrose synthesized by Sun et al. [77]. It was reported that pressing temperature of more than 170 °C led to certain extent of dry bond strength where 190 °C resulted in the best adhesive curing. Plywood pressed at 190 °C has a wet shear strength that surpassed the China National Standard GB/T 9846-2015. Nevertheless, the boards pressed at 130 and 150 °C exhibited no adhesion strength, implying that the CA/sucrose adhesive did not cure well at low temperature. Pressing time also plays an important role in the curing of CA/sucrose adhesive where 7 min was reported to the most optimum parameter. The higher the glue spread rate, the higher the shear strength of the plywood. Therefore, a glue spread rate of 140 g/m^2^ is sufficient as the wear shear strength of the plywood produced has already surpassed the China National Standard GB/T 9846-2015 and therefore application of higher glue spread rate is not necessary. To conclude, to fabricate CA/sucrose-bonded plywood with acceptable performance, one has to: (1) synthesize the CA/sucrose adhesive with ratio of 75/25 (CA/sucrose) at 100 °C for 2 h, and (2) hot-press the plywood at 190 °C for 7 min using a glue spread rate of 140 g/m^2^. Further study on reducing the pressing temperature and time is current underway.

## 4. Comparison between CA-Treated Particleboard with Conventional Particleboard

Data listed in Table 4 compare the mechanical and physical properties of the CA-bonded particleboard with other particleboard bonded with conventional adhesives. It can be seen the performance of CA-bonded particleboard is comparable to that of conventional particleboard. However, higher density (800 kg/m^3^) and resin content (20 wt %) are needed for CA-bonded particleboard to attain that comparable strength. It is noted that new giant reed particleboard (700 kg/m^3^) bonded with 5 and 10 wt % CA has very low MOR and MOE as well as IB [72] compared to that of 10 wt % UF-bonded poplar particleboard with the same density [82]. Particleboard bonded with pMDI required a relatively lower resin content (8 wt %) to attain suffice strength performance, followed by PF which is 12 wt %. At 20 wt %, CA-bonded particleboard exhibited higher MOE and lower TS value than both pMDI- and PF-bonded particleboard [67]. It is interesting to note that CA-bonded particleboard has better dimensional stability than pMDI- and PF-bonded, which indicated by lower TS value after 24 h soaking. Therefore, it can be concluded that CA showed promising potential in replacing conventional adhesive to produce particleboard with satisfactory properties.

## 5. Environmental Impact and Future Outlook

### 5.1. Environmental Impact of CA-Treated Wood

Modifying wood with CA, a green renewable chemical, is expected to be able to enhance the performance of a product and reduce the final environmental footprint. Logically, modified wood is expected to possess longer service life expectancy compared to that of the untreated wood and subsequently lower maintenance requirements [18]. This is owing to the fact that modified wood is normally bestowed with enhanced technological performance [91], reduced hygroscopicity, and improved durability after treatment [91,92]. There is only one study regarding the environment impacts of CA-treated wood found. Essoua et al. [93] studied the environmental impacts of CA and glycerol treated Lodgepole pine wood siding and the damage impact categories was evaluated using life cycle assessment (LCA). After treatment with CA, the treated siding has 2.8 times longer life expectancy than the untreated ones. For land occupation and impact for respiratory organics categories, CA-modified siding exhibited 80% and 44% lower impact, respectively, compared to that of untreated siding. Overall, CA-treated wood siding gives more impact to the environment than the untreated siding.

When simplified into five simple stages, which is production, distribution, installation, maintenance and end of the life, the production stage of treated wood siding presented the most environmental impacts, followed by maintenance stage. The production and maintenance stage respectively contributed 88.2% and 11.8%. Energy and material consumed in the CA production is one of the important factors that contributing to this finding. On the contrary, 99.9% of the total resources impact in untreated wood siding are contributed by the production stage where 82.9% comes from timber processing including extraction, sawing, drying and planning. It should be noted that all of the assessment made is based on the scenario where both treated and untreated has a service life expectancy of 20 years. It is reported that CA-modified wood would become more ecofriendly than untreated wood siding only when the service life expectancy of the treated wood siding reaches 55 years, which is five times that of untreated wood siding [93]. Therefore, future study should be focused on identifying low environmental impact treatments.

### 5.2. Future Outlook

Due to the rapid growth of food additives in the processed food industry, in the year of 2014, the global market of CA has reached USD 2.6 billion. It is expected to achieve USD 3.6 billion by the year of 2020 with a projection compound annual growth rate (CAGR) rate of 5.5%. The largest share in the market are dominated by European region in the year of 2014 as a result of flourishing processed food and beverage market. Food industry is still the leading industry in the application of CA [94]. However, the application of CA in the wood-based industry is believed to be expanded in the near future had its potential been fully excavated. One of the driven factors is the environmental issue. Environmental awareness among the consumers increased continuously in the recent years as a response to the climate policy actions [95]. At the same time, industries have been urged for adapting a transformation from a waste intensive linear economy system toward a less waste-producing and longer lifetime production-consumption system due to the emergence of so-called circular economy policy [96,97]. One of the corresponding strategies that needed to be taken by the industries is to produce products with longer durability and better recyclability [98]. Modified wood automatically becomes the target as it would influence the recyclability of the wood and subsequently its environmental footprint. Nevertheless, studies on the particular topic in assessing the possibilities and challenges in recycling of modified wood is relatively scarce.

Modified wood products have become popular since the 2000s. Criticism received for the toxicity nature of traditional preservative treatment in combination with the dwindling sources of naturally durable tropical hardwoods has diverted the world’s attention to modified wood. Up until now, the role of modified wood has been insignificant in the circular economy. However, Sommerhuber et al. [99] believed that its market share will be expanded. Subsequently, the needs for recycling modified wood will also increase. Therefore, the task in ensuring the recovered raw materials and subsequent products free from any toxic chemical are most vital. All of this favours the application of a green modifying agent such as CA. 

### 5.3. Economic Perspective

From the aspect of green binder for wood composite, the application of CA is expected to face some challenges as CA is strongly dependent on its market price. The price of commercialized CA is ranging from USD 0.70 to USD 2.00 per kilogram [100]. CA production was once a lucrative sector. Unfortunately, the high cost of raw materials and energy has changed the scenario in the recent years. If this situation persisted, it will hinder the willingness of manufacturers to adopt CA as a replacement for conventional wood adhesive. Therefore, seeking a more economical substrate or processing pathway is an urgent priority in order to reduce the cost of production [101].

## 6. Conclusions

CA has been studied and recognized as an environmentally friendly compounds to improve some selected properties of the modified wood. Apart from that, CA has also been proven able to substitute synthetic resin as a binding agent for the manufacturing of wood-based panels. The application of CA as a modifying agent and binder for wood has been reviewed in this paper. The following summaries can be drawn based on the review of the present literatures and works:

1. Wood modified with CA displayed an improvement in MOE, compression strength and dimensional stability and biological resistance as well as reduced WA.
2. Glycerol, glucose, and sorbitol can be combined with CA to resulted in a better treatment.
3. Several factors have been identified as able to affect the effectiveness of CA treatment on wood, namely catalyst type, wood species, and curing temperature. Apart from SHP, chlorhydric acid (HCl) and para-toluenesulphonic acid (p-TSA) are also among the most effective catalyst. A temperature of at least 140 °C is required to ensure the polyesterification occurs to a greater extent.
4. In the fabrication of wood-based composites, CA alone could attain sufficient bonding properties for the resultant boards. However, sucrose and starch could be added to improve some selected properties of the boards.
5. For the manufacturing of particleboard, particleboard with the optimum properties could be attained when 20 wt % of CA/sucrose adhesive with ratio of 25/75 (CA/sucrose) was pressed at a temperature >180 °C for 10 min, with the condition that pre-drying treatment of 12 h at 80 °C was adopted.
6. Contrarily, bonding the flat surfaces involves a completely different application compared to particleboard and fiberboard. Therefore, to fabricate CA/sucrose-bonded plywood with acceptable performance, one has to: 1) synthesize the CA/sucrose adhesive with ratio of 75/25 (CA/sucrose) at 100 °C for 2 h, and 2) hot-press the plywood at 190 °C for 7 min using a glue spread rate of 140 g/m^2^.
7. One of the challenges faced by CA is that it requires a higher pressing temperature and longer pressing time. Further study should be conducted to match the industrial practice.

## Figures and Tables

**Table 1:** Advantages and disadvantages of CA as wood treating agent.

Advantages	Disadvantages
Reduction in water absorption (WA)	No improvement in weathering resistance
Improved resistance against termites and fungi	Reduction in modulus of rupture (MOR)
Improved modulus of elasticity (MOE) and compression strength	Reduction in impact strength
Better dimensional stability	Yellowing of the treated wood

**Table 2:** Types materials source and products produced by using citric acid as bonding agent.

Materials Source	Products	Adhesive Solution	Reference
*Acacia mangium* bark powder	Wood based molding	Citric acid	[57]
*Acacia mangium* bark powder	Wood based molding	Citric acid	[60]
Bamboo materials	Particleboard	Citric acid	[61]
Teak	Particleboard	Critic acid and sucrose	[62]
Recycled wood particles	Particleboard	Citric acid and sucrose	[63]
Recycled wood particles	Particleboard	Citric acid and sucrose	[64]
Petung (*Dendrocalamus asper*)	Particleboard	Citric acid and starch	[65]
Sweet sorghum bagasse	Particleboard	Citric acid	[66]
Sweet sorghum bagasse	Particleboard	Citric acid	[67]
Recycled wood particles	Particleboard	Tannin, sucrose and citric acid	[68]
Nipa fronds	Particleboard	Maltodextrin, sucrose and citric acid	[69]
Nipa fronds	Particleboard	Maltodextrin and citric acid	[70]
alang-alang (*Imperata cylindrica*)	Particleboard	Citric acid	[71]
New Giant Reed (*Arundo Donax* L.)	Particleboard	Citric acid	[72]
Sugarcane bagasse	Particleboard	Citric acid and sucrose	[73]
Pineapple (*Ananas comosus* (L.) Merr.) leaves	Medium density fiberboard	Citric acid and sucrose	[74]
Elephant dung fibers	Fiberboard	Citric acid	[75]
Poplar (*Populus tomentosa* Carr)	Plywood	Citric acid and sucrose	[76]
Poplar (*Populus tomentosa* Carr)	Plywood	Citric acid and sucrose	[77]
Poplar Veneer	Wood veneer panels	Citric acid	[58]

**Table 3:** Factors influencing the properties of CA-bonded particleboard.

Studies	Reference
**Board density**	
Bending strength of particleboard increased proportionately as the density increased from 400 kg/m^3^ to 800 kg/m^3^ and maintained an almost constant value at 1000 kg/m^3^. 800 kg/m^3^ is the optimum density for particleboard. TS increased with increasing density but maintained at <12%. WA decreased as the board density increased. Board density of 800 kg/m^3^ is required to obtained optimum water resistance.	[64]
**Press temperature and time**	
Bending strength was low when pressed at 140 and 160 °C, but increased significantly when pressed at 180 °C and remained constant at temperature above 180 °C. The internal bonding (IB) strength increased significantly when pressed at temperature up to 200 °C and started to decreased when pressed at 220 and 240 °C. A press temperature of 200 °C or higher is required to obtain optimum dimensional stability.	[64]
The IB and bending strength of the bamboo particleboard increased along with pressing temperature and the maximum IB was recorded when 200 °C was used and decreased sharply when 220 °C was used. TS and WA displayed the same trend. The IB and bending strength of the bamboo particleboard increased when the pressing time increased from 2 to 10 min and started to decreased when pressed at 15 min. TS and WA decreased along with increasing pressing time. No significant different was detected between pressing time of 10 and 15 min. A pressing time of 10 min is optimum for particleboard production	[67]
**Pre-drying treatment**	
Pre-drying treatment time of 6 h and 12 h at 80 °C has reduced the TS from 26.1% to 13.3% and 7.7%, respectively. MOR, MOE and IB of the particleboard produced was higher when pre-drying treatment of 12 h was applied compared to that of without pre-drying treatment or after 6 h pre-drying treatment.	[71]
Particleboard made from pre-dried particles at 80 °C for 12 h has higher bending strength.	[66]
**Citric acid content**	
Particleboard bonded with 20 wt % CA has significantly lower TS compared to that of the particleboard bonded with 10 and 15% CA.	[71]
Bending strength of the particleboard increased as the CA content increased from 0 to 20 wt % but decreased when 30 wt % were applied. As for IB strength, 30 wt % resulted in the best result. TS and WA decreased along with increasing CA content.	[66]
The most optimum IB value was obtained when the particleboard was bonded with 15 wt % CA. Any addition of CA content did not bring significant improvement in IB.	[62]

**Table 4:** Comparison between citric acid bonded particleboard and particleboard bonded with conventional adhesive.

Materials	Density (kg/m^3^)	Type of Resin	Resin Content (wt %)	MOR (MPa)	MOE (MPa)	IB (MPa)	TS (%)	Reference
Recycled wood particles	800	Citric acid	20	10.7	3300	0.32	-	[63]
	800	Citric acid: sucrose (25:75)	20	20.1	4400	1.13	-	
Sweet sorghum bagasse	800	Citric acid	20	21.8	5200	0.89	10.1	[67]
	800	PF	12	32.9	4500	0.78	20.6	
	800	pMDI	8	34.1	4600	1.33	23.1	
New Giant Reed	700	Citric acid	5	3.91	1019	0.27	38.99	[72]
	700	Citric acid	10	4.99	1245	0.22	28.03	
Rubberwood	700	MUF	8	14.3	2152	1.3	38.2	[83]
Oil palm trunk	700	UF	8	11.18	1843	1.01	14.99	[84]
Poplar	700	UF	10	14.57	2015	1.32	31.26	[82]
Eucalyptus	720	pMDI	4	10.4	1651	1.45	26.95	[85]
Date palm trunk	700	PF	10	18	2970	0.95	17.6	[86]
	700	MUF	10	17.6	2890	0.9	19.8	
Beech	720	UF	8	16.3	3261	-	18.2	[87]
Pine	720	UF	8	10.3	1913	0.51	64.71	[88]
*Acacia mangium*	730	MDI	12	15.2	-	0.8	-	[89]
Eucalyptus	620	UF	10	17.1	2869	0.4	15.6	[90]
Pine	620	UF	10	13.6	2450	0.47	10	
Sugarcane bagasse	620	UF	10	15.3	2295	0.26	11.6	
